# Nutritional and Chemical Composition and Antiviral Activity of Cultivated Seaweed *Sargassum naozhouense* Tseng et Lu

**DOI:** 10.3390/md11010020

**Published:** 2012-12-27

**Authors:** Yan Peng, Enyi Xie, Kai Zheng, Mangaladoss Fredimoses, Xianwen Yang, Xuefeng Zhou, Yifei Wang, Bin Yang, Xiuping Lin, Juan Liu, Yonghong Liu

**Affiliations:** 1 Key Laboratory of Marine Bio-resources Sustainable Utilization, Guangdong Key Laboratory of Marine Materia Medica, RNAM Center for Marine Microbiology, South China Sea Institute of Oceanology, Chinese Academy of Sciences, Guangzhou 510301, China; E-Mails: py00_2006@126.com (Y.P.); moses10c@gmail.com (M.F.); xwyang@scsio.ac.cn (X.Y.); xfzhou@scsio.ac.cn (X.Z.); binggo525@163.com (B.Y.); xiupinglin@hotmail.com (X.L.); ljuan2010@qq.com (J.L.); 2 Fisheries College, Guangdong Ocean University, Zhanjiang 524025, China; E-Mail: xieenyi@163.com; 3 Guangzhou Jinan Biomedicine Research and Development Center, Jinan University, Guangzhou 510632, China; E-Mails: twangyf@jnu.edu.cn (K.Z.); twangyf@jnu.edu.cn (Y.W.)

**Keywords:** *Sargassum naozhouense*, seaweed, chemical composition, antiviral activity

## Abstract

*Sargassum naozhouense* is a brown seaweed used in folk medicine and applied for thousands of years in Zhanjiang, Guangdong province, China. This study is the first time to investigate its chemical composition and antiviral activity. On the dry weight basis, this seaweed was constituted of *ca.* 35.18% ash, 11.20% protein, 1.06% lipid and 47.73% total carbohydrate, and the main carbohydrate was water-soluble polysaccharide. The protein analysis indicated the presence of essential amino acids, which accounted for 36.35% of the protein. The most abundant fatty acids were C14:0, C16:0, C18:1 and C20:4. The ash fraction analysis indicated that essential minerals and trace elements, such as Fe, Zn and Cu, were present in the seaweed. IR analysis revealed that polysaccharides from cultivated *S. naozhouense* may be alginates and fucoidan. The polysaccharides possessed strong antiviral activity against HSV-1 *in vitro* with EC_50_ of 8.92 μg/mL. These results demonstrated cultivated *S. naozhouense* has a potential for its use in functional foods and antiviral new drugs.

## 1. Introduction

Seaweeds, classified into red algae (Rhodophyta), brown algae (Ochrophyta, Phaeophyceae) and green algae (Chlorophyta) [1,2], are a renewable natural resource with extensive distribution along the Pacific coast [3]. They have been used mainly for human consumption (e.g., as food or as crude drugs to treat gallstone, stomach ailment, eczema, cancer, renal disorders, scabies, psoriasis, asthma, arteriosclerosis, heart disease, lung diseases and ulcers) and extraction of hydrocolloids, such as agar, carrageens and alginates, but are still under-exploited [3,4,5,6,7,8,9,10]. In recent years, seaweeds have caused emerging interest in biomedicine and the food area, because they possess a wealth of bioactive compounds (such as sulfated polysaccharides, carotenoids, dietary fiber, protein, essential fatty acids, vitamins, minerals, terpenoids, oxylipins, phlorotannins and steroids) with potential industrial and agricultural applications [11,12,13,14,15,16]. For example, alginates from brown algae are often used as additives to ameliorate the texture of food [7]. Therefore, seaweeds are a promising renewable resource with considerable commercial potential for further exploitation. 

Brown seaweeds (e.g., *Sargassum fusiforme* and *Saccharina japonica*—formerly *Laminaria japonica*) have been used as Traditional Chinese Medicines in China for thousands of years [17,18]. *Sargassum naozhouense *Tseng et Lu, an edible brown algae widely distributed along the coasts of Zhanjiang, Guangdong province, China, is commonly consumed as a sea vegetable or as crude drugs for treating internal heat, infections, laryngitis and other ailments in locals [19]. According to literature reports, the wild *S. naozhouense *is rich in polysaccharides, amino acids and trace elements [19]. However, little is known about the cultivated *S. naozhouense*, especially its nutritional and functional properties. For full utilization of this rich resource, it is imperative to evaluate the nutritional and functional properties of the cultivated *S. naozhouense*. In general, the nutritional properties are usually estimated by the chemical composition. Moreover water-soluble sulfated polysaccharides are the main constituents of seaweed cell walls, with potent antiviral activities, particularly against HSV [20,21]. Therefore, the aim of this study was to investigate the chemical composition of cultivated *S. naozhouense* and the anti-HSV activity of water-soluble polysaccharide from cultivated *S. naozhouense*.

## 2. Results and Discussion

### 2.1. Chemical Composition of Cultivated *S. naozhouense*

The ash, protein and total carbohydrate were the most abundant constituents in *S. naozhouense* (Table 1). The average contents of protein and ash were 11.20% and 35.18% in dry weight, respectively, which were close to those reported for the wild *S. naozhouense* (13.95% and 41.79%) [19] and higher than those of *Saccharina japonica* (8.70% and 20.00%) [22]. Furthermore, the protein content was comparable to that recorded for some species of the same genus, *i.e.*, *S. henslowianum* (11.52%) and *S. fusiforme *(15.38%) [23,24].

**Table 1:** Chemical composition of cultivated *S. naozhouense* (%, w/w on the dry basis) ^a^.

Components	Values
Ash	35.18
Protein	11.20
Lipid	1.06
Total carbohydrate	47.73
Total water-soluble carbohydrate	29.74
Water-soluble polysaccharide	21.01
Total dietary fiber	4.83

^a^ Average of four analyses.

Interestingly, the total carbohydrate level (47.73%) was higher than that reported for *S. fusiforme* (46.01%) and wild *S. naozhouense* (29.37%) [19,24]. Moreover, the main carbohydrates were water-soluble polysaccharides (21.01%), yet the dietary fiber content (4.83%) was relatively lower. On the other hand, the lipid content (1.06%) was relatively lower. This result was similar to that of wild *S. naozhouense* (2.4%) and other edible brown algae, such as *Saccharina japonica* (0.2%) and *S. fusiforme *(0.69%) [22,24]. 

### 2.2. Amino Acid Composition

The amino acid composition of proteins in cultivated *S. naozhouense* was illustrated (Table 2). The contents of amino acids ranged from 0.54 to 13.21 g/100 g protein. The proteins of cultivated *S. naozhouense* contained a high level of amino acids, especially essential amino acids (EEA), e.g., leucine (6.52 g/100 g protein) and valine (4.64 g/100 g protein). Furthermore, all essential amino acids, such as valine, methionine, isoleucine, leucine, phenylalanine, lysine, histidine, arginine and tryptophan, accounting for 47.22% of the total amino acids, were present in this seaweed. The ratio value of EAA/NEAA and the essential amino acid index (EAAI) were 0.89 and 66.24, respectively. According to FAO/WHO recommended standards of ideal protein [25], the protein of cultivated *S. naozhouense* belongs to a high-quality protein. Furthermore, the protein quality is better than that of *S. fusiforme*, because cysteine is lacking in *S. fusiforme* [24]. 

In addition, the aspartic (8.39 g/100 g protein) and glutamic acids (13.21 g/100 g protein), non-essential amino acids (NEEA), were the most abundant amino acids and accounted for 28% of total amino acids, which, together with glycine (4.38 g/100 g protein) and alanine (5.27 g/100 g protein), were responsible for the special flavor and taste of cultivated *S. naozhouense*.

**Table 2:** Amino acid composition of cultivated *S. naozhouense *(g/100 g protein) ^a^.

Amino acids	Contents	Amino acids	Contents
Aspartic acid	8.39	Tyrosine	2.95
Threonine	3.93	Phenylalanine	4.38
Serine	3.21	Histidine	1.07
Glutamic acid	13.21	Lysine	3.66
Proline	3.30	Arginine	4.20
Glycine	4.38	Tryptophan	0.89
Alanine	5.27	Total	76.97
Valine	4.64	EAA	36.35
Methionine	2.41	NEAA	40.62
Cysteine	0.54	EAA/NEAA	0.89
Isoleucine	4.02	EAAI	66.24
Leucine	6.52		

^a^ Average of four analyses; EAA: essential amino acids, Threonine, Valine, Methionine, Isoleucine, Leucine, Phenylalanine, Lysine, Histidine, Arginine, and Tryptophan; NEAA: non-essential amino acids; EAAI: essential amino acid index.

### 2.3. Fatty Acid Composition

The fatty acid composition of cultivated *S. naozhouense* is presented (Table 3). This seaweed contained high concentrations of saturated fatty acids (SAFA, 33.63% of total of fatty acid), monounsaturated fatty acid (MUFA, 10.42% of total of fatty acid), and polyunsaturated fatty acid (PUFA, 18.84% of total of fatty acid), even though it had a low level of lipid. 

The main fatty acids in cultivated *S. naozhouense* were C14:0 (myristic acid), C16:0 (palmitic acid), C18:1 (oleic acid) and C20:4 (arachidonic acid), which were also the most abundant fatty acids in edible seaweed *S. fusiforme* [24]. However, C16:0 (palmitic acid), C18:0 (stearic acid) and C18:1 (oleic acid) were the most abundant fatty acids in wild *S. naozhouense* [19]. 

**Table 3:** Fatty acid composition of cultivated *S. naozhouense* (% of total of fatty acid) ^a^.

Fatty acids	Methyl esters (%)	Fatty acids	Methyl esters (%)
C6:0	0.44	C18:3ω3	0.25
C8:0	0.49	C20:1	0.53
C12:0	0.34	C20:3ω6	2.95
C14:0	6.7	C20:4ω6	9.61
C15:0	0.3	C20:5ω3	1.38
C16:0	24.61	C22:0	0.21
C16:1	3.55	SAFA	33.63
C16:2ω6	0.22	MUFA	10.42
C18:0	0.54	PUFA	18.84
C18:1	6.34	PUFAω6	13.24
C18:2trans	3.97	PUFAω3	1.63
C18:2ω6cis	0.46	Ratioω6/ω3	8.12

^a^ Average of four analyses; SAFA: saturated fatty acids; MUFA: monounsaturated fatty acids; PUFA: polyunsaturated fatty acids.

Although our study revealed that cultivated *S. naozhouense* had higher total levels of PUFA than MUFA, the eicosapentaenoic acid (EPA, C20:5ω3) and essential fatty acids, such as C18:2ω6cis (linoleic acid), C18:3ω3 (linolenic acid, and C20:4ω6 (arachidonic acid), the most interesting and important fatty acids in terms of nutrition, were present in this seaweed. Further, the ratio of ω6/ω3, which the WHO currently recommends should not be higher than 10 in diet as a whole [26], was 8.12, which indicated the cultivated *S. naozhouense* may be used as a sea vegetable or an ingredient to reduce ω6/ω3 ratio in diet.

### 2.4. Mineral Contents

Different mineral elements (such as potassium, sodium, phosphorus, calcium, iron, zinc, manganese, copper and cadmium) were analyzed by inductive coupled plasma-optical emission spectroscopy (ICP-OES) and were summarized (Table 4). The cultivated *S. naozhouense* contained significant amounts of essential minerals (e.g., potassium, sodium, calcium and phosphorus), like *S. fusiforme* and wild *S. naozhouense* [19,24]. Potassium (4170 mg/100 g dry weight) was the most abundant element in the seaweed, followed by sodium (3250 mg/100 g), phosphorus (120 mg/100 g) and calcium (66.98 mg/100 g). The ratio of Na/K (0.77) was relatively lower, which was interesting from the point of view of nutrition, because high Na/K ratio diets and the incidence of hypertension are closely connected [27]. Consequently, the cultivated *S. naozhouense* may be useful for the regulation of the Na/K ratio of diets.

On the other hand, cultivated *S. naozhouense* also contained trace elements, such as iron, manganese, zinc, copper and cadmium. Iron was the most abundant trace element (147 mg/100 g), followed by Zn (9.08 mg/100 g). The content levels of other trace elements (Table 4) were similar to those recorded in the earliest reports on seaweeds [15,28]. Furthermore, the contents of some heavy metal elements (As, Cd, Cu, Hg and Pb) in this seaweed were below the toxic limits allowed in some countries [29]. Hence, cultivated *S. naozhouense* may be used as a food supplement to provide the daily intake of some trace elements (e.g., iron, zinc) for adults, especially iron, since iron deficiency would lead to anemia, when the demand for iron is high in growth, high menstrual loss and pregnancy [27].

**Table 4:** Mineral composition of cultivated *S. naozhouense* (mg/100 g) ^a^.

Minerals	Contents
K	4170
Na	3250
P	120
Ca	66.98
Fe	147
Zn	9.08
Mn	5.84
Cu	0.36
Cd	0.17

^a^ Average of four analyses.

### 2.5. Properties of Polysaccharide

**Figure 1:**
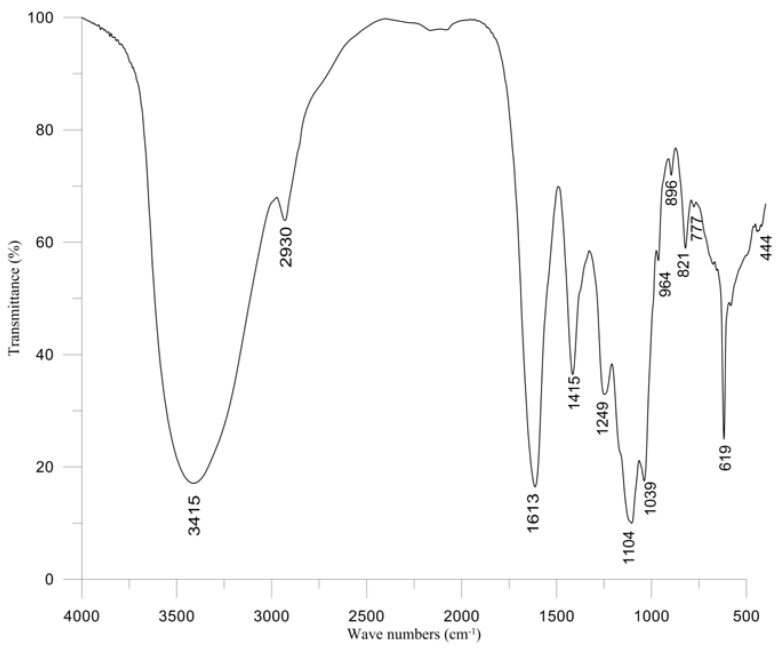
FT-IR spectrum of water-soluble polysaccharides from cultivated *S. naozhouense*.

The content of water-soluble polysaccharides from cultivated *S. naozhouense* was 21.01% (Table 1). The IR spectrum of polysaccharides was recorded in a potassium bromide pellet using an IR spectrophotometer. In the IR spectrum (Figure 1), it is being observed that a broad peak at 3415 cm^−1^ and a small peak at 2930 cm^−1^ are due to the stretching vibrations of O–H and C–H, respectively. The bands at 1613 and 1415 cm^−1^ were attributed to carboxylate O–C–O asymmetric stretching and to C–OH deformation vibrations, respectively. The absorption at 1039 cm^−1^ was assigned to C–O and C–C stretching vibrations of the pyranose ring. The anomeric region of the fingerprint (950–750 cm^−1^) exhibited three characteristic absorption bands in alginate polysaccharides (Figure 1, bands at 896, 821 and 777 cm^−1^, respectively). The band at 896 cm^−1^ is assigned to the β-anomeric C–H deformation vibration of β-mannuronic acid residues. The band at 821 cm^−1^ seems to be characteristic of mannuronic acid residues. The band at 777 cm^−1^ is assigned to gluluronic acid [30]. In addition, a broad band at 1249 cm^−1^ indicated the presence of sulphated ester groups (S=O), which is a characteristic component in fucoidan [31,32,33]. Therefore, the water-soluble polysaccharides from cultivated *S. naozhouense* may be alginates and fucoidan, which should be further demonstrated by more studies in the future.

### 2.6. Cytotoxic and Antiviral Activities of the Polysaccharides

The polysaccharides exhibited lower cytotoxicity on Vero cells (CC_50_, MCC > 200 μg/mL). After Vero cells had been treated by various polysaccharide dilutions for two days, cell morphology did not have any visible alteration under a phase-contrast microscope, and cell layer also did not have any destruction by MTT reduction assay.

The polysaccharide showed strong antiviral activity against HSV-1 strain F at ≥12.5 μg/mL (EC_50_ = 8.92 μg/mL). In order to compare antiviral potential of the polysaccharides, acyclovir (ACV) was used as a positive control and conferred more than 75% cellular protection at 20 μg/mL, which was in agreement with that the polysaccharides at 12.5 μg/mL (Table 5). Moreover, the selectivity index (SI, CC_50_/EC_50_), which was often higher than 10 for a sample with antiviral activity [7], was more than 22. It is clear that water-soluble polysaccharides from cultivated *S. naozhouense* possess anti-HSV-1 activity *in vitro*.

Herpes simplex virus type 1 (HSV-1), a common human pathogen, is responsible for a broad range of human infectious diseases. Though ACV is served as a drug to successfully treat the HSV infections, ACV-resistant strains have been found in immune-compromised patients and drug toxicity has also been reported [34,35]. Therefore, searching for new antiviral agents is urgently needed. Natural bioactive compounds are the best resources for the development of new anti-HSV drugs due to their greater efficiency with less toxicity. Sulfated polysaccharides from marine algae, including sulfated mannans, galactans, agarans, fucoidans, sulfated rhamnogalactans, fucans and different types of carrageenans, were showed to be active against some enveloped viruses, especially HSV and HIV [21,36,37,38,39]. In this study, the sulfated water-soluble polysaccharides showed strong antiviral activity against HSV-1 strain F *in vitro* within noncytotoxic concentration. Consequently, the sulfated polysaccharides from marine algae are a good resource for searching novel therapeutic candidates for HSV.

**Table 5:** Effect on HSV-1strain F replication in Vero cells and cytotoxicity of polysaccharide from cultivated *S. naozhouense*.

**Polysaccharide**	**Concentration (μg/mL)**	**100**	**50**	**25**	**12.5**	**6.25**	**3.12**	**1.56**	**0.78**	**Virus contrast**
	CPE	–	–	–	+	++++	++++	++++	++++	++++
	EC_50_ (μg/mL)	8.92								
	CC_50_ (μg/mL)	>200								
	MCC (μg/mL)	>200								
**Acyclovir (ACV)**	Concentration (μg/mL)	20								
	CPE	+								

“++++”: >75% cytopathic; “+++”: 50%–75% cytopathic; “++”: 25%–50% cytopathic; “+”: 0%–25% cytopathic; “–”: no cytopathic effect induced by virus.

## 3. Experimental Section

### 3.1. Algal Material

The cultivated *S. naozhouense *was collected from Techeng Island, Guangdong province of China in July 2011 and identified by Professor Weixin Li and Dr. Xie Enyi, Fisheries College, Guangdong Ocean University, China. A voucher specimen (No. P110701) was deposited in the Key Laboratory of Marine Bio-resources Sustainable Utilization, South China Sea Institute of Oceanology, Chinese Academy of Sciences, Guangzhou, China. The fresh seaweeds were washed in freshwater to remove sediment, epifauna and epiphytes, and then dried in the air for 10 h, powdered and stored in plastic bags at 4 °C until further experiment use.

### 3.2. Chemical Composition

Total nitrogen was quantified by the Kjeldahl method, and then, the protein content was estimated by multiplying the total nitrogen content by a nitrogen conversion factor of 6.25 [8]. Total ashes were determined by incinerating seaweed samples in a digitally controlled furnace with temperature being gradually increased to 550 °C and maintaining for 6 h, and then were quantified gravimetrically [40]. Total lipids, total water-soluble carbohydrates and total dietary fibers were determined by the Soxhlet method, anthrone-sulfuric acid colorimetry and the gravimetric method, respectively [40]. Total carbohydrate was determined by the difference method [24]. Polysaccharide content was estimated by the phenol-sulfuric acid method, using glucose as a standard substance [41]. Amino acids were determined by high-performance liquid chromatography (HPLC) according to the GB/T 5009.124-2003 standard method [42]. Fatty acid composition was determined by gas chromatography-mass spectrometry (GC-MS) analysis of their methyl esters on a Varian Gas Chromatograph series 3800 fitted with a VF-5 MS fused silica capillary column (30 m × 0.25 mm, film thickness 0.25 μm, USA) [43]. Mineral analysis was made by inductive coupled plasma-optical emission spectroscopy (ICP-OES) [43].

### 3.3. Preparation of Polysaccharides

Approximate 20.0 g seaweed powder were accurately weighed and defatted in a Soxhlet apparatus with petroleum ether (60–90 °C), then pretreated twice with 80% ethanol to remove some pigments, monosaccharides, oligosaccharides and other small molecule materials. After the organic solvent was volatilized, the pretreated seaweed powder was extracted twice with distilled water at 90 °C for 1.5 h and filtered. The combined aqueous extracts were concentrated in a rotary evaporator to a certain volume under reduced pressure at 50 °C, followed by treatment with Sevag Reagent to remove protein and centrifugation at 5000 rpm for 20 min to obtain the supernatant. Then the supernatant was poured into six volumes of 100% ethanol and was kept at 4 °C overnight. The precipitate containing crude polysaccharides was collected by centrifugation, then washed with 70% ethanol, 100% ethanol, ethyl ether and acetone, respectively, and finally, freeze-dried, weighed and kept in a vacuum dryer [44,45,46].

### 3.4. FT-IR Spectroscopy

Infrared spectra were recorded from a KBr pellet of the polysaccharide on a spectrometer FT-IR Nicolet 6700.

### 3.5. Determination of Antiviral Activity of the Sulfated Polysaccharide

#### 3.5.1. Cells and Virus

African green monkey kidney cells (Vero, ATCC CCL-81), provided by Wuhan Institute of Virology, Chinese Academy of Sciences, were cultured in Dulbecco’s modified Eagle medium (DMED, Invitrogen) supplemented with 10% FBS (Invitrogen), 0.22% sodium bicarbonate (Sigma) and 50 mg/L gentamycin (Invitrogen). HSV-1 strain F (ATCC VR733), obtained from Hong Kong University, was propagated in Vero cells and stored at −80 °C until use.

#### 3.5.2. Cytotoxicity Assay

Cytotoxicity of the polysaccharides on Vero cells was evaluated *in vitro* by MTT assay as described by Mosmann [47]. Vero cells (1 × 10^4^ cells/well) were seeded in 96-well plates and incubated at 37 °C in 5% CO_2_ atmosphere for 24 h. Then, various dilutions (concentration from 3.12 to 200 μg/mL) of polysaccharide were added to wells, with quadruplicate wells for each concentration, and further incubated for 48 h; meanwhile, cells were examined daily under a phase-contrast microscope to determine the minimum concentration of polysaccharide (MCC) that caused a microscopically detectable alteration of cell morphology. Afterwards, MTT solution was added (final concentration 0.5 mg/mL) to each well. After 4 h of incubation at 37 °C, the supernatant was removed, and the dimethyl sulfoxide (DMSO) was added to solubilize the formazan crystals, then the optical density (OD) was measured in a microplate reader at 570 nm. The cytotoxicity was expressed as 50% cytotoxic concentration (CC_50_), which was the concentration of the test substances required to reduce cell growth by 50%.

#### 3.5.3. Antiviral Activity Assay

The antiviral activity of the polysaccharide was evaluated by cytopathic effect (CPE) inhibition assay [48]. In general, Vero cells were seeded in 96-well plates at a density of 1 × 10^4^ cells per well and allowed to form a monolayer. The confluent cell monolayer was treated with serial two-fold dilutions of polysaccharide and an equal volume of virus suspension (100TCID_50_) in quadruplicate in 96-well plates and then incubated at 37 °C in a 5% CO_2_ atmosphere and observed daily for CPE under a light microscope. Meanwhile, acyclovir (ACV) was served as a positive control. The 50% effective antiviral concentration (EC_50_), defined as the concentration that reduced CPE by 50% with respect to the virus control, was calculated by MTT method.

## 4. Conclusions

The edible cultivated brown algae *S. naozhouense* was investigated for its potential nutritional value and antiviral activity for the first time. It is characterized by a high level of proteins and a low level of lipid, like *S. fusiforme* and *Saccharina japonica* used in human nutrition [18] and, therefore, can be used for human consumption as an alternative source of essential amino acids and some polyunsaturated acids, such as oleic, linoleic, linolenic and eicosapentaenoic acids, or as functional ingredients to reduce calories and modify the texture of formulated foods. This seaweed is also rich in some minerals, such as iron and zinc, and so may be used as a food supplement to supply these minerals at low inclusion levels. Especially, the protein in cultivated *S. naozhouense* was better than that in *S. fusifome*, because cystein was present in the former. In this regard, cultivated *S. naozhouense* has higher nutritional value compared to *S. fusiforme*, which has been consumed as a longevity sea vegetable in Chinese traditional diets and may be a better alternative resource of the Traditional Chinese Medicine *S. fusifome*.

On the other hand, cultivated *S. naozhouense* has a high level of water-soluble polysaccharides, which is very interesting, because pharmacological and biological activities of polysaccharides from marine algae in therapeutic applications for humans are already well-known [49]. Furthermore, the water-soluble polysaccharides contained sulfate groups and exhibited strong antiviral activity against HSV-1 strain F *in vitro* with an EC_50_ of 8.92 μg/mL and a SI of >22. Hence, the polysaccharide from cultivated *S. naozhouense* has a potential in antiviral new drugs. Further studies should be carried out for the isolation, characterization and other biological screening of the polysaccharide. 

In summary, the brown alga *S. naozhouense *may represent an interesting advance in the search for novel functional applications in relevant industrial uses, including pharmaceuticals, nutraceuticals, cosmeceuticals and functional foods. 
